# The cotton swab method: an accurate and less invasive way to assess fecal consistency in weaned pigs

**DOI:** 10.1186/s12917-024-03888-1

**Published:** 2024-02-03

**Authors:** Esben Østergaard Eriksen, Martin Friis Sejersen, Ken Steen Pedersen

**Affiliations:** 1 Department of Veterinary and Animal Sciences, Section for Production Nutrition and Health, University of Copenhagen, Grønnegårdsvej 2, 1870 Frederiksberg C, Denmark; 2 Ø-Vet, Køberupvej 33, 4700 Næstved, Denmark; 3 The Faculty of Veterinary Medicine, Department of Production Animal Clinical Sciences, Norwegian University of Life Sciences, Elizabeth Stephansens vei 15, 1433 Ås, Norway

**Keywords:** Fecal consistency, Clinical score, Stool scale, Fecal dry-matter, Pig, Diarrhea, Fecal score

## Abstract

**Background:**

Researchers and pig veterinarians are interested in assessing pigs’ fecal consistency. This study developed a standardized protocol and scale for the cotton swab method, which is a way of assessing the fecal consistency in pigs. The accuracy of the cotton swab method was evaluated in weaned pigs using fecal dry-matter analysis as a golden standard. The study also proposed fecal dry-matter percentage thresholds for the categorization of fecal consistency on a four-point scale.

**Results:**

The thresholds of 10.3%, 16.6%, and 21.9% fecal dry-matter were suggested for categorization of the consistency of fecal samples on a four-point scale. The accuracy of the cotton swab method was high. The agreement to the four-point fecal consistency score derived from the fecal dry-matter percentage was almost perfect (weighted Gwet’s agreement coefficient = 0.87 [95% confidence interval: 0.84; 0.91]). The cotton swab method had a sensitivity of 85.0% (95% confidence interval: 76.5; 91.4) and a specificity of 95.2% (95% confidence interval: 92.0; 97.3) when used to diagnose whether pigs had diarrhea or not. For non-diarrheic pigs, the method almost always (n = 287/289) required less handling than the collection of a fecal sample by digital rectal manipulation.

**Conclusion:**

The cotton swab method is an accurate way to assess fecal consistency in pigs, both on a four-point scale and as a dichotomous diarrhea score. The method is quick to perform and less invasive than methods relying on the collection of fecal samples. New fecal dry-matter thresholds between feces of different consistencies were proposed.

**Supplementary Information:**

The online version contains supplementary material available at 10.1186/s12917-024-03888-1.

## Introduction

In both experimental and observational studies, researchers are interested in assessing weaned pigs’ fecal consistency. For instance, trials investigating the effect of different interventions on gut health in weaned pigs often record the fecal consistency as a measure of diarrhea severity, and such studies are published at a high rate [1, 2]. Field studies also characterize whether pigs suffer from diarrhea or not and relate this to other factors, e.g., the presence of microorganisms; however, the method for fecal consistency assessment is often not reported [3]. For practical purposes, for instance, in diagnostic investigations or for systematic monitoring, herd personnel and veterinarians are also interested in assessing the fecal consistency of weaned pigs. At least four methods have been described for fecal consistency assessment: fecal dry-mater analysis, visual inspection of fecal samples, assessing diarrheic soiling of the hind part, and the cotton swab method.

Fecal dry-matter analysis estimates the percentage of dry-matter in a fecal sample [4, 5]. This method provides objective measures on a discontinuous scale (percentage), and it can be viewed as a golden standard test since it has a high repeatability when more than 1 g of feces is used [5]. However, there has been some disagreement on which thresholds to apply when interpreting the results [4–6].

The fecal consistency may also be determined by visual inspection. A clinician judges the consistency on a validated clinical scale, or binary as diarrhea/not diarrhea [5, 7]. Scales with different levels or categories have been described [2], including three [8, 9], four [5, 7] or five levels [10, 11]. The major weakness of these methods is that they require a fecal sample. In clinical trials with single animal housing and frequent monitoring of the pigs (e.g., [11]), fecal samples may be assessed when the pigs defecate spontaneously. However, in many applications (e.g., [6, 12]) samples are obtained by stimulating the pig to defecate by digital rectal manipulation. This requires time and resources, and it may cause stress and discomfort for the pigs. Furthermore, some pigs will not deliver a fecal sample [2], and this causes a problem with missing values not at random; diarrheic pigs are less reluctant to deliver a sample than non-diarrheic pigs [6]. In addition, fecal dry matter analysis requires time and resources for the laboratory analysis. Hence, the method is mainly relevant for certain research applications.

The fecal consistency can also be predicted based on the presence/absence of diarrheic soiling of the hind part [13]. However, this method only allows imperfect dichotomous (diarrhea/not diarrhea) predictions, and the diagnostic sensitivity is only acceptable during the first 14 days after weaning, making the method invalid in older pigs [12–14].

In at least two published studies, the cotton swab method has been used to assess the fecal consistency in pigs [15, 16]. The method is simple. Using a cotton swab, a small sample of feces is collected directly from the rectum of the pig of interest, and the clinician evaluates the consistency of the sample as it appears on the cotton swab. The studies have simply categorized pigs as diarrheic or non-diarrheic [15, 16]. Hypothetically, the method might also be able to categorize consistency on a four-point scale. The method potentially overcomes the problem of missing values and may possibly require less handling of the pigs than the collection of full fecal samples. However, the method has not been validated.

Therefore, the aim of this study was to validate the cotton swab method for fecal consistency assessment in weaned pigs. The study did this by pursuing four specific objectives, which were:

1. to develop a standardized protocol for the cotton swab method and a scale capturing the four previously defined fecal consistencies [7];
2. to evaluate the accuracy of the cotton swab method when using it to categorize fecal consistency on a four-point scale;
3. to evaluate the accuracy of the cotton swab method when using it to dichotomously categorize fecal consistency as non-diarrhea or diarrhea;
4. and, as a prerequisite for objectives 1–3, to define thresholds to be used when assigning fecal consistency based on fecal dry-matter estimates.

## Results

### Fecal-dry matter thresholds

Table 1 displays thresholds for fecal dry-matter percentages discriminating four previously defined fecal consistencies [5, 7]. Between firm (score 1) and soft and shaped (score 2) feces, 24.2% and 23.0% were equally good thresholds, so a compromise of 23.6% was selected.

**Table 1:** Thresholds for fecal dry-matter percentage defining four previously described fecal consistencies

**Fecal consistency: Qualitative description**	**Current study**	**Pedersen et al. **[5]	**Eriksen et al. **[6, 13]	**Total (mean)**
Firm	> 23.6^a^	> 19.5	> 22.7	21.9
Soft and shaped	≤ 23.6^a^	≤ 19.5	≤ 22.7	≤ 21.9
Loose (diarrhea)	≤ 16.8	≤ 18.0	≤ 15.0	≤ 16.6
Watery (diarrhea)	≤ 10.4	≤ 11.3	≤ 9.0	≤ 10.3

Thresholds were established based on data from the scale validation phase of the current study compared to previously published thresholds, and a compromise (the crude mean) was suggested. The consistencies were first defined by Pedersen et al. [5, 9]

^a^24.2% and 23.0% were equally good thresholds, so a compromise of 23.6% was selected

### Protocol and scale development

The protocol and scale development phase resulted in the standardized protocol for the cotton swab method described in Table 2. A preliminary scale was also proposed, and this is enclosed in Additional File 1.

**Table 2:** How to use the cotton swab method: A five-step protocol

Step	Description
1	Hold the cotton swab, leaving approximately 25–30 mm of stick between the cotton tip and your pinched fingers
2	Slowly insert the cotton swab 25–30 mm into the rectum of the pig, i.e., corresponding to where you hold the swab
3	Tilt the swab slightly at a 10–15° angle to the cranio-caudal axis of the rectum. Rotate the swab three times clockwise around the cranio-caudal axis of the rectum at this angle. Thereafter, rotate the swab three times counter-clockwise at a 10–15° angle. Do not roll the swab around its own axis/between your pinched fingers
4	Retract the swab from the rectum
5	Assess the fecal consistency as it appears on the cotton swab immediately (within five seconds). If the pig defecates in response to the procedure, the fecal consistency of the delivered fecal sample should be taken into consideration in your assessment

#### Scale adjustment

**Fig. 1:**
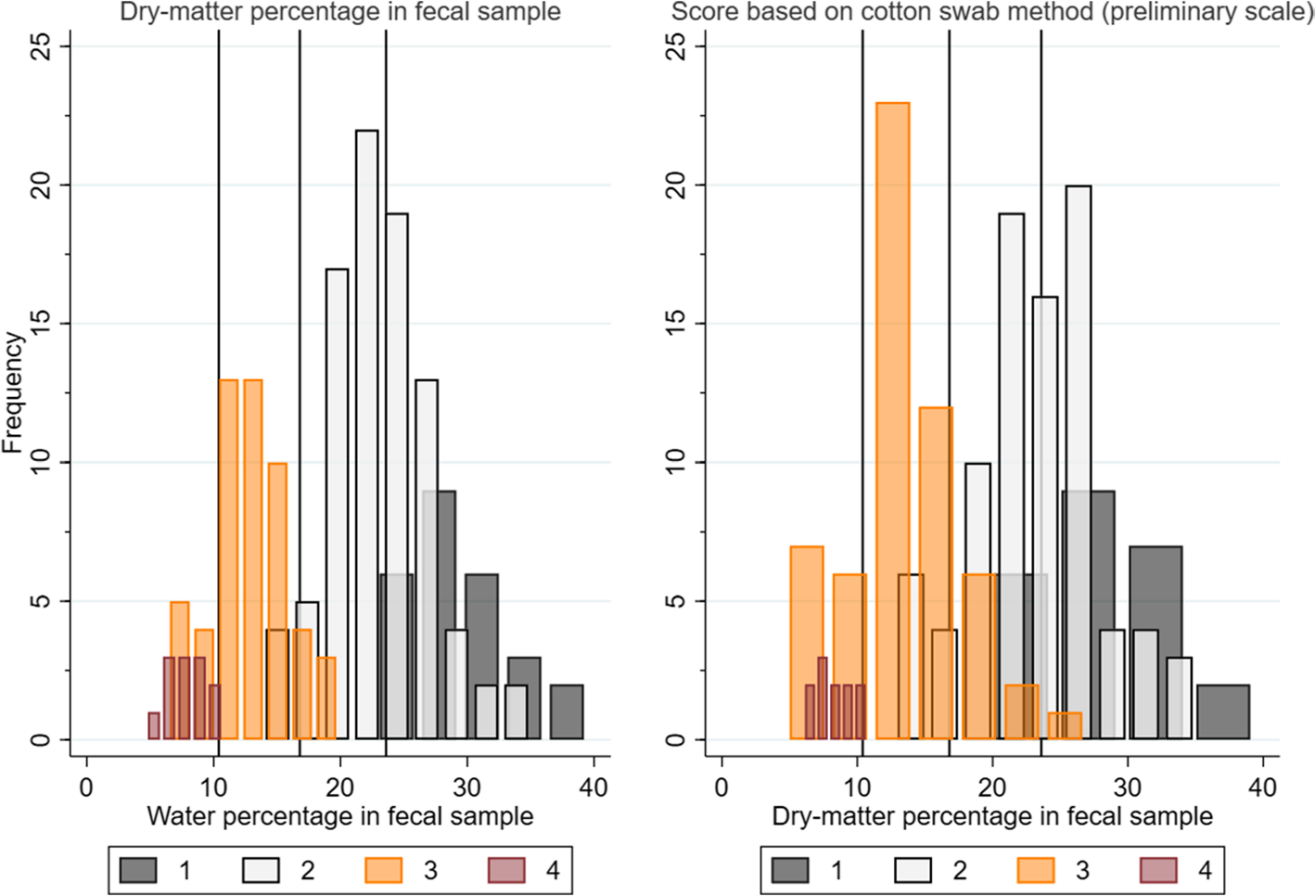
Frequencies of consistency scores from the scale adjustment phase plotted against the fecal dry-matter percentage. Histograms of the scores of fecal consistency given by visual inspection of fecal samples (left panel) and the cotton swab method using the preliminary scale (right panel) in the scale adjustment phase. Reference lines (x = 23.6, 16.8, 10.4) display empirical thresholds between the four feces consistencies (see Table 1)

In the scale adjustment phase, the preliminary scale (Additional File 1) was evaluated. Cotton swab scores and fecal samples were successfully collected from 178 pigs. The fecal dry-matter percentage and the fecal sample consistency score (1–4) are displayed in Fig. 1 (left panel). The data indicated that the clinical observer had applied an erroneous interpretation of the scale for consistency evaluation based on visual inspection of fecal samples. Specifically, the fecal dry-matter content was too high for score 1, *i.e.,* only very firm/dry feces were given the score 1. Accordingly, the samples given the score 2 often included dry-matter percentages expected to be given the score 1. This systematic misclassification error was also reflected in the criteria described for scores 1 and 2 in the preliminary scale (Additional File 1). It may be acknowledged from Fig. 1 (right panel), displaying the fecal dry-matter percentage and the cotton swab fecal consistency score (1–4). Based on these findings, the scale for assessment when using the cotton swab methods was adjusted, and this final proposal is displayed in Table 3.

**Table 3:**
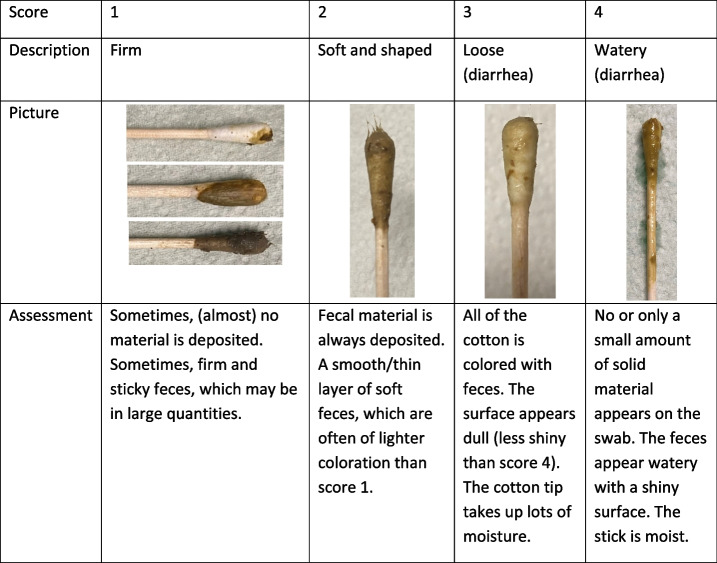
How to score the fecal consistency on a four-point scale using the cotton swab method

### Scale validation

In the scale validation phase, complete registrations were collected from 212 pigs.

#### Assessment on a four-point scale

**Fig. 2:**
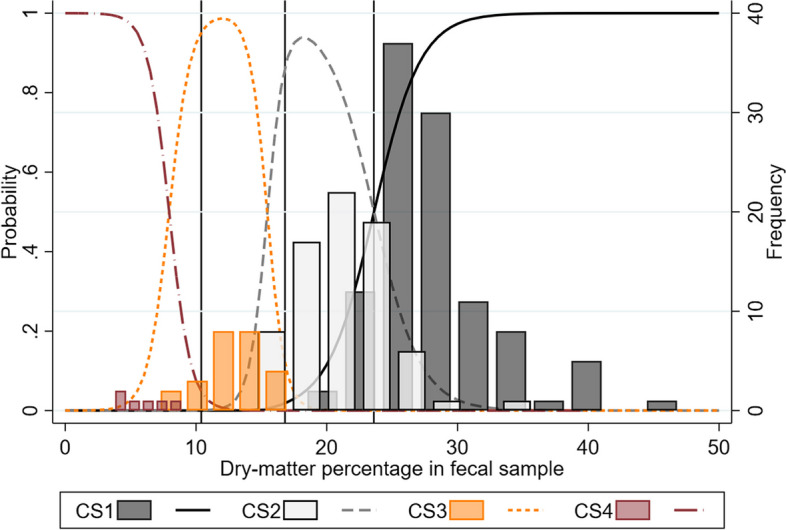
The probabilities and frequencies of cotton swab scores plotted against the fecal dry-matter percentage. The predicted probability of a pig being given a certain consistency score using the cotton swab method and histograms displaying the frequency of scores 1–4, both as a function of the dry-matter percentage in a fecal sample collected from the pigs (n = 212). Reference lines (x = 23.6, 16.8, 10.4) display empirical thresholds between the four feces consistencies (see Table 1)

Figure 2 displays histograms with frequencies of the cotton swab fecal consistency scores (1–4) divided by the fecal dry-matter percentage. In addition, Fig. 2 includes predicted probabilities of being given a fecal consistency score with the cotton swab method as a function of the dry-matter percentage. The unweighted overall agreement between the fecal dry-matter-based consistency scores (1–4) and the cotton swab consistency scores (1–4) was 0.79, and the weighted agreement was 0.93. The unweighted Gwet’s agreement coefficient (AC) was 0.74 (95% CI: 0.67; 0.81), and the weighted Gwet’s AC was 0.87 (95% CI: 0.84; 0.91), indicating an almost perfect agreement [17–19].

**Fig. 3:**
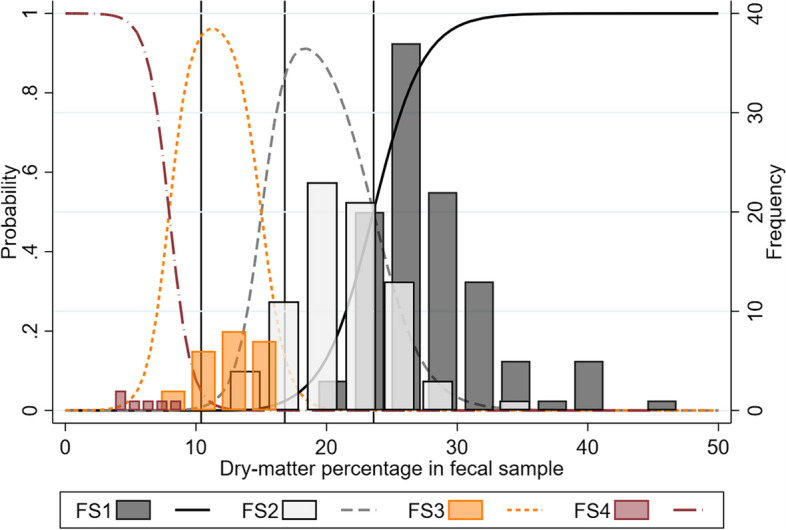
The probabilities and frequencies of consistency scores for visual assessment of fecal samples plotted against the fecal dry-matter percentage. The predicted probability of a pig being given a certain consistency score using visual inspection of a fecal sample and histograms displaying the frequency of scores 1–4, both as a function of the dry-matter percentage in a fecal sample collected from the pigs (n = 212). Reference lines (x = 23.6, 16.8, 10.4) display empirical thresholds between the four feces consistencies (see Table 1)

For comparison, Fig. 3 displays histograms with the frequencies of the fecal consistency scores (1–4) obtained by visual inspection of fecal samples plotted against the fecal dry-matter percentage. The unweighted agreement was 0.78, and the weighted agreement was 0.93. The unweighted Gwet’s AC was 0.73 (95% CI: 0.66; 0.80) and the weighted Gwet’s AC was 0.87 (95% CI: 0.83; 0.90) for the consistency scores based on visual inspection of a fecal sample.

#### Dichotomous assessment

The threshold between scores 2 and 3, i.e., diarrhea and not diarrhea, was not changed between the preliminary scale (Additional File 1) and the final scale (Table 3). Therefore, when dichotomizing the assessment from the cotton swab method, the data from the scale adjustment phase and the scale validation phase could be merged and analyzed together.

Dichotomous assessments (diarrhea/not diarrhea) based on the cotton swab method had a diagnostic sensitivity of 85.0% (95% CI: 76.5; 91.4) and a specificity of 95.2% (95% CI: 92.0; 97.3). The corresponding two-by-two table is displayed in Table 4.

**Table 4:** Cotton swab method diagnosis of pigs with or without diarrhea

	CS pos	CS neg	Total
Diarrhea	85	15	100
Not diarrhea	14	276	290
Total	99	291	390

CS pos.: Cotton swab positive, i.e., diagnosed as diarrheic

CS neg.: Cotton swab negative, i.e., diagnosed as non-diarrheic

The true diarrhea status was based on fecal dry-matter analysis (golden standard), using 16.6% dry-matter as the cut-off

For comparison, the visual inspection of a fecal sample yielded a diagnostic sensitivity of 87% (95% CI: 78.8; 92.9) and a specificity of 97.9% (95% CI: 95.6; 99.2).

#### Defecation in response to the cotton swab method

Performing the cotton swab method, i.e., handling the pig and inserting the cotton swab into the rectum as described in Table 2, sometimes stimulated the pigs to defecate. The deposited feces should be taken into account in the consistency evaluation (Table 2, Step 5). As seen from Table 5, having diarrhea, especially watery diarrhea, had a clear effect on the probability of defecating in response to the procedure.

**Table 5:** Occurrence of defecation in response to the cotton swab method divided by fecal consistencies

Dry-matter based fecal consistency	n/N	Predicted % (95% CI)
Firm	1/217	0.3 (0.04; 2.4)
Soft and shaped	1/72	0.8 (0.09; 6.2)
Loose (diarrhea)	9/64	12.6 (6.2; 23.9
Watery (diarrhea)	19/27	66.8 (42.0; 84.8

The table displays the frequency and predicted percentage of pigs defecating in response to handling and performance of the cotton swab method; the measures are divided by the fecal consistency category assigned based on fecal dry-matter analyses. The predicted percentage of defecation was derived from a logistic model adjusting for the herd and age of the pigs

## Discussion

### Thresholds for categorization of consistency based on fecal dry-matter

As a prerequisite for our main objectives, we determined thresholds for the reference test we wanted to validate against, i.e., consistency classification based on fecal dry-matter analysis. We estimated thresholds according to a four-point scale [7] and compared the results to previously suggested thresholds [5, 6]. The studies did not agree completely on the thresholds. This may be explained by inter-observer variation in the interpretation of the scale [7, 9] and random variation. Furthermore, fecal specimens from different ages were used. Eriksen et al. [6] assessed samples collected during the first week after weaning. Pedersen et al. [5] used samples from pigs later in the nursery phase. The present study analyzed pigs aged up to five weeks after weaning. It is possible that the same fecal dry-matter content may appear visually different depending on the age of the pigs. We suggest that the common thresholds proposed in Table 1 may be adopted in future studies of fecal samples from weaned pigs. It should be noted that previous research projects have used other clinical definitions of diarrhea (i.e., slightly firmer or more watery), and those data indicated that 20% [4, 8] or 12% [20] dry-matter were good cut-offs between diarrhea and not diarrhea.

### Accuracy of the cotton swab method

There was an almost perfect agreement between the score given when assessing cotton swabs according to the final scale (Table 3) and the golden standard, the fecal dry-matter based consistency score. The diagnostic sensitivity and specificity for the dichotomous assessments were high, but not perfect. From Fig. 2, it may be construed that the false positives and false negatives will likely be soft and shaped feces tending to be loose, or loose (diarrheic) feces tending to be soft and shaped. That is, the incorrectly classified pigs will often be in the periphery between diseased and non-diseased. In most applications, both practical and in research, it will be less detrimental to misclassify such cases than cases that are severely diarrheic or clearly non-diarrheic.

The measures of accuracy were almost identical for the assessments based on visual inspection of fecal samples. In a previous study, farmers and veterinarians classified pictures of fecal pools from nursery pigs as diarrheic or not. Compared to the present study, similar diagnostic sensitivities (0.83 and 0.85) but slightly lower specificities (0.86 and 0.89) were reported [21]. In conclusion, the accuracy of cotton swab method is high, and it seems to perform just as well as assessments based on visual inspection of a fecal sample.

A limitation of the study is that the sample size was small. Especially the number of pigs with consistency scores of 3 or 4 was limited in the scale validation phase. Hence, the measures of accuracy were associated with some uncertainty, which was described by the confidence intervals. Yet, these were small enough to make it unlikely that the true accuracy of the cotton swab method is less than good.

The reproducibility, i.e., the intra-observer and inter-observer agreement, of the cotton swab method remains to be assessed. Intra-observer and inter-observer variation of at least the same magnitude as for assessments based on visual inspection of fecal samples [7] may reasonably be expected.

To our knowledge, the use of the cotton swab method was first described in suckling piglets [16]. In this age group, obtaining a fecal sample by digital rectal manipulation may be even more difficult and invasive than in weaned pigs, making the method tempting to use. However, it should be noted that the present validation was performed on weaned pigs, and hence the accuracy may be different for suckling pigs. Furthermore, the criteria used for classification could likely be different when assessing feces from piglets primarily living on a milk-based diet. Moreover, it may be relevant to determine how the creamy/pasty diarrhea, typically associated with coccidiosis [22], presents on a cotton swab. Future studies may also evaluate to what extent the cotton swab method allows detection of deposits, such as mucus, necrotic debris, or blood, as these sometimes occur in samples of post-weaning diarrhea [6].

As such, the cotton swab method may be readily adopted in research and clinical practice for weaned pigs. It is advisable to train the use of the scale and to evaluate the reproducibility associated with the specific observer(s).

### Feasibility of the cotton swab method

This study did not evaluate the feasibility of the cotton swab method empirically. However, our experience from the present study and a cohort study involving more than 3000 clinical examinations [15] is that the method is easy to use. The predicted probabilities in Table 5 indicate that for non-diarrheic pigs, the cotton swab method will almost always entail less handling than the collection of fecal samples by digital rectal manipulation. Hence, the cotton swab method is a less invasive procedure, and it also supports the hypothesis that the collection of fecal samples may result in values missing not-at-random [6]. Both of these conclusions favor the use of the cotton swab method compared to methods relying on the collection of a fecal sample. The results in Table 5 also indicate that defecating in response to handling or insertion of the cotton swab may in itself be a sign of diarrhea.

### Statistical considerations

Inappropriate statistical methods are often applied to fecal consistency scores in porcine research [2]. The cotton swab method uses a four-point scale; it is not a continuous scale, and one can hardly argue that a distribution with four levels is Gaussian (normal). Therefore, statistical approaches such as estimating the mean, performing an analysis of variance (ANOVA) test, or fitting a linear regression will, in many instances, be inappropriate. The data should be treated as ordinal or nominal. If one uses the cotton swab method to measure *the fecal consistency*, the four-point scale is truly ordered, with score 1 being less than score 2, score 2 less than score 3, and score 3 less than score 4. However, if the *extent/severity of diarrhea* is the measure of interest, as it commonly is, it may actually be more appropriate to view the scale as nominal. This is because the scores 1 and s 2 represent the same level of diarrhea, namely “not diarrhea.” Hence, if one ranks the extent of diarrhea, it seems erroneous to assume that pigs with score 1 have less diarrhea than pigs with score 2. Collapsing the scores 1 and 2 into one category may allow to analyze the data as ordinal.

## Conclusions

The cotton swab method produces accurate assessments of the fecal consistency both on a four-point scale and dichotomously (diarrhea/not diarrhea). The method has similar accuracy to visual inspection of fecal samples, and it is quicker to perform and less invasive than the collection of fecal samples by rectal stimulation. New fecal dry-matter thresholds between feces of different consistencies were proposed.

## Materials and methods

The work was conducted in September and October 2021, structured in three phases:

1. Protocol and scale development
2. Scale adjustment
3. Scale validation

**Fig. 4:**
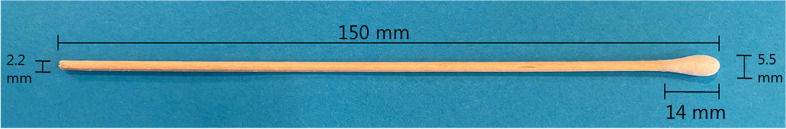
Picture of the used cotton swab (Producer: DANSU A/S) with measures

Throughout the three phases, data was collected by the same observer (the author, MFS) in three conveniently sampled indoor pig herds (herds A, B, and C) located in Jutland, Denmark. Herd A had between 1100 and 1200 sows (the exact number is not provided for GDPR-reasons), herd B had between 800 and 900 sows, and herd C had between 400 and 500 sows. When approximately 3–4 weeks old, pigs were weaned into segregated climate-controlled nursery units with semi-slatted floors, either on the same site as the sow unit (herds B and C) or at different sites (herd A). Medicinal zinc oxide was given in the feed the first 14 days after weaning in all herds. Throughout the study, we used the cotton swab shown in Fig. 4. According to the producer (DANSU A/S), the stick was produced in white birch wood, and the head contained 0.04 (± 10%) grams of cotton.

### Protocol and scale development

During two visits in herd A, we performed an explorative, iterative process. The appearance of the feces on a cotton swab was evaluated, and at the same time, a fecal sample was collected from the pigs by digital rectal manipulation. Hereby, we developed a standardized protocol for the cotton swab method, and a preliminary scale with four levels was suggested.

#### Scale adjustment

Data was collected in a cross-sectional design during two herd visits at herds A and B, respectively. In sections with pigs weaned within the last five weeks, a number of pigs were selected by systematic random sampling, as shown in Table 6. We used the procedure for systematic random sampling described in Additional File 2 of Eriksen et al. [6] with minor modifications.

**Table 6:** Number of pigs included in the scale adjustment phase and the scale validation phase

Herd	Batch age	Scale adjustment	Scale validation
Herd A	1	24	12
	2	24	12
	3	24	12
	4	24	12
	5	24	0
Herd B	1	29	12
	2	29	12
	3	0	12
	4	0	12
Herd C	1	0	29
	2	0	29
	3	0	29
	4	0	29
Total		178	212

Pigs were sampled from three herds, herds A, B, and C, in two work phases: the scale adjustment phase and the scale validation phase. Batch age is measured in weeks since weaning/insertion into the nursery unit

The variables described in Table 7 were collected from all pigs. First, the cotton swab method was performed. A score was assigned according to the preliminary scale (see Additional File 1). If a given pig had not defecated in response to the cotton swab method, digital rectal manipulation was used to stimulate the pig to defecate. We were patient with pigs that did not respond to digital rectal manipulation and waited for them to defecate in order to avoid missing values. Fecal samples were collected in plastic containers [23]. The consistency of the fecal samples were scored at the herd visit, and afterwards the samples were stored at -20 °C until fecal dry-matter analysis.

**Table 7:** Variables collected from each pig in the scale adjustment phase and the scale validation phase

Variable	Description
Fecal sample collection	A binary variable with the following levels: 0: Fecal sample delivered immediately in response to the handling of the pig or the insertion of the cotton swab into the rectum 1: Fecal sample obtained by digital rectal manipulation
Cotton swab consistency score (1–4)	Assessment of the fecal consistency on a four-point scale according to either the preliminary scale (Additional File 1) in the scale adjustment phase or the final scale (Table 3) in the scale validation phase
Cotton swab diarrhea score	A dichotomization of Cotton swab fecal consistency score (see above). Where 1 and 2 were considered not diarrhea, 3 and 4 were considered diarrhea
Fecal sample consistency score (1–4)	Assessment of fecal consistency on a four-point scale based on visual inspection of a fecal sample [7]: 1, Firm; 2, Soft and shaped; 3, Loose (diarrhea); 4, Watery (diarrhea)
Fecal sample diarrhea score	A dichotomization of Fecal sample consistency score (1–4) (see above). Where 1 and 2 were considered not diarrhea, 3 and 4 were considered diarrhea
Fecal dry-matter %	The percent of the fecal sample that was dry-matter (in opposition to water)
Dry-matter based consistency score (1–4)	The true fecal consistency based on the fecal dry-matter percentage using the thresholds shown in the “Total” column in Table 1
Dry-matter based diarrhea score	A dichotomization of Dry-matter based consistency score (1–4) (see above). Where 1 and 2 were considered not diarrhea, 3 and 4 were considered diarrhea

The data was analyzed, and, based on our findings, the scale was adjusted to increase the agreement with fecal-dry matter analyses of the fecal samples.

### Scale validation

In the scale validation phase, data was collected in a cross-sectional design. Pigs were selected by systematic random sampling, as shown in Table 6. Four herd visits were performed; herd C was visited twice. The variables collected (Table 7), the clinical examinations, and the processing of fecal samples were performed as in the scale adjustment phase, with the modification that we used the final scale (see Table 3) that was proposed as a result of the scale adjustment phase.

### Fecal dry-matter analysis

All containers were weighed (precision 0.01 g) without the lid prior to sample collection and again with fecal content prior to drying. The containers with the whole sample were dried in an oven at 70 C° until constant weight (> 12 h). The samples were placed in a desiccator while cooling to avoid uptake of atmospheric humidity, and then weighed again. The fecal dry-matter percentage (FDM%) was then estimated as:

$$FDM\mathrm{\%}=\frac{a-c}{b-c}*100$$

where *a* denotes the weight of the container and sample *after* drying; *c* denotes the container weight; and *b* denotes the weight of the container and sample *before* drying. The lightest fecal sample collected weighed 2.55 g, and therefore problems with large measurement errors for samples less than 1 g [5] were not relevant to consider.

### Statistical analyses

Data was collected on paper sheets and typed into MS Excel 2016 [24]. All the statistical work was conducted in Stata IC 16 [25]. Simple descriptive statistics were used to get an overview of the two datasets.

#### Fecal dry-matter thresholds between consistencies

Using the consistency assessment and fecal dry-matter of fecal samples from the scale validation phase, we suggested thresholds for a four-level categorization of fecal samples based on fecal dry-matter % as previously described [5]. We harvested similar thresholds from two studies [5, 6] and estimated the unweighted means between the studies to propose a common set of thresholds.

#### Evaluating the accuracy for the cotton swab method

The accuracy of the four-point scale for cotton swab consistency assessment was evaluated using the data from the scale validation phase. The overall agreement and Gwet’s AC [26] with linear weights were estimated [18] to assess the agreement between the dry-matter based consistency score (1–4) and the cotton swab consistency score (1–4). Gwet’s AC is a measure of agreement similar to the commonly used Cohen’s kappa statistic. We chose this statistic because the prevalence of the diarrheic samples, especially with score 4, was low in our study population. Gwet’s AC adjusts for the effect of prevalence in the test population, which the Kappa statistic is sensitive to, and minimizes the bias introduced when the proportions of each score are dissimilar between tests [27]. Gwet’s ACs were interpreted as proposed by Landis and Koch [17], with the modification that assignment to the benchmarking intervals should consider that estimates of coefficients of agreement have a probability distribution [18, 19]. A multinomial model was fitted with the cotton swab consistency score (1–4) as the outcome and the fecal dry-matter percentage as a predictor. Marginal mean predictions were made by applying an inverse log transformation to linear predictions from the model with the command written by Pitblado [28]. For comparison, similar estimations (overall agreement, Gwet’s AC, and multinomial model) were carried out for fecal scores obtained by visual inspection of a fecal sample [9].

The accuracy of dichotomous assessments was evaluated using the data from both the scale adjustment phase and the scale validation phase. This was appropriate since the criteria discriminating score 3 from score 2 (i.e., diarrhea or not-diarrhea) were not changed between the preliminary scale and the final scale. The diagnostic sensitivity and specificity were estimated [29] for the dichotomous assessment of the fecal consistency based on the cotton swab method. For comparison, the same statistics were estimated for visual inspection of a fecal sample.

#### Estimating the causal effect of fecal consistency on defecation

We estimated the causal effect of fecal consistency on the risk of defecating in response to handling and the cotton swab method. A logistic model was fitted with the variable *Fecal sample collection* (Table 7) as the outcome and the variable *Dry-matter based consistency score (1–4)* (Table 7) as a fixed effect. Lack of independence and possible confounding effects were adjusted for by adding *Herd* and *Batch age* (Table 6) as fixed effects. We assumed that no other variables affected the relation between fecal consistency and the risk of defecation and that the handling procedure was independent (i.e., performed similarly) of the fecal consistency. Hence, the estimated association would represent the average direct effect [30]. Marginal mean predictions were made as described for the multinomial models.

### Supplementary Information

**Additional file 1.** Preliminary scale for the cotton swab method. The preliminary scale that was first proposed but found to be erroneous and therefore adjusted in the scale adjustment phase.

**Additional file 2.** Dataset the cotton swab method. The dataset supporting the conclusions of the present paper.

## Data Availability

The dataset supporting the conclusions of this article is included as an additional file.

## Abbreviations

CI: Confidence interval
Gwet’s AC: Gwet’s agreement coefficient
