# Supplementary material for: The cotton swab method: an accurate and less invasive way to assess fecal consistency in weaned pigs
Source: BMC Vet Res. 2024 Feb 3;20:47. doi: 10.1186/s12917-024-03888-1 (PMC10837864; doi:10.1186/s12917-024-03888-1)
Supplement: Supplementary file 1 — Additional file 1. Preliminary scale for the cotton swab method. The preliminary scale that was first proposed but found to be erroneous and therefore adjusted in the scale adjustment phase. [file 12917_2024_3888_MOESM1_ESM.pdf]

Score	1	2	3	4
Description	Firm	Soft and shaped	Loose (diarrhea)	Watery (diarrhea)
Picture	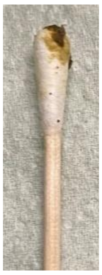	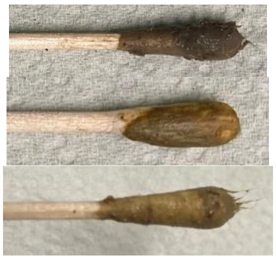	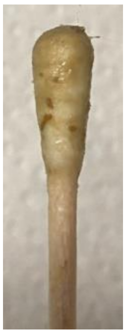	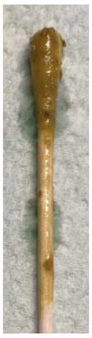
Assessment	Slight amount of material deposited.	A thick layer of fecal material is sticking to the swab.	All of the cotton is colored with feces. The surface appears dull (less shiny than score 4). The cotton tip takes up lots of moist.	No or only a small amount of solid material will appear on the swab. The feces will appear watery with a shiny surface. The stick is moisty.
